# Extra-Limites

**Published:** 1836-10-01

**Authors:** 


### EXTRA-LIMITES.

### DR. RYAN'S FORMULARY OF HOSPITALS.

*To the Editors of the Medico-Chirurgical Review.*

Gentlemen,—I have seen in your last number, a condemnatory notice by an anonymous correspondent, of my translation of " A New Practical Formulary of Hospitals, &c." I was the more surprised at this, knowing you had previously pronounced the following opinion of the work.—" This is one of the most valuable little vade-mecums that we have ever seen. It is indispensable for the practitioner of every grade and denomination."—*Medico-Chirurgical Review, October*, 1835.

In nine months after the date just given, and when a second edition of the work is announced, you think it " justice to the public," to allow some anonymous scribbler, to give the most unqualified contradiction to your own opinions; and to occupy three pages in vilifying and misrepresenting the work you so highly praised.

It is clear, that if your opinion of the book be correct, that of your correspondent must be false or malicious, as it is totally opposite to your own.

It is not usual, so far as I know, for reviewers to allow their criticisms to be questioned, or their decisions to be reversed; indeed I have never before known an instance of the kind. You pronounce the Formulary of Hospitals the best you have ever seen, while your candid critic declares, " it is, without exception, one of the most inaccurate, and, consequently, useless publications ever presented to the medical public." What a specimen of impartial British reviewing is this, and in the same journal too! It is time to expose this system as the bane of medical literature.

Permit me to observe, that I have often received the severest strictures on works of which I had conscientiously given a favourable opinion; but I considered such communications an insult to my judgment, and only worthy of a fiery ordeal. They were generally the effusions of spleen, or dictated by some unworthy motive; and among them were strictures on some of the senior Editor's own productions, as severe and unjust as those of which I now complain; and I am by no means certain, that some of them were not in the hand-writing of your correspondent and critic T. P. I need not observe that there are envious and disappointed spirits in our profession, who are ever ready to fling dirt at those, whose talents and acquirements, have placed them high in public estimation.

It is much to be regretted, that nearly all our medical periodicals, are too ready to admit such reviews, and nearly all are influenced by mean, party and paltry motives, which are most injurious to the advancement of science. After long observation and ample evidence of the workings behind the scenes, I have come to the conclusion, that by far the greatest portion of medical criticism in this kingdom is beneath the notice of any man of an independent mind, or of standing in the profession. You belong to the honourable few to whom these remarks do not apply, and yet no doubt through accident or inadvertency, you allow your pages to be defiled by an interested and unprincipled writer.

Before I offer a few words in reply to this considerate critic, permit me to allude to the other notices " of the most useless book ever published," in his opinion!!

The British and Foreign Medical Review has strongly recommended the work; and even the Medical Gazette, the most unfair and dishonest review ever published, in any country, after a vast deal of abuse (for it has invaribly abused all my works, and those of every one else who is not of its clique, notwithstanding the favourable criticisms of the respectable journals both at home and abroad), designated the Formulary of Hospitals " a Polyglot Pharmacopæia." That " veracious and trustworthy critic," has predicted the failure of all my works, every one of which has been republished abroad, and passed through different editions at home. He was most furious a few months since on the appearance of a new edition of my Jurisprudence, he pronounced it the worst book ever published, because at that moment, his employers were reprinting, without note or comment, the last American edition of Dr. Beck's most comprehensive work on the same subject! I allude to these facts to prove the truth of a former statement; to expose the comtemptible conduct of some of our Medical Reviewers, and to shew them how much I fear their criticism.

But unfair criticism is most injurious to authors and to medical literature. An author publishes a work after many years research and observation, at an expense by no means trifling; and the moment it appears, it is abused because he does not belong to a certain faction in the profession, or because he did not give the work to a certain publisher. This is our happy system of reviewing and encouraging medical authors. The sale of their works is injured or destroyed, and they are many pounds sterling out of pocket! But if works possess intrinsic merit, they cannot be suppressed by venal and hireling critics, among whom stands your correspondent.

Of all the incompetent and ignorant critics I have met, this one is the most perfect example, as I shall prove, I think, to the conviction of your readers.

He commences by charging me with the typographical blunders, every one of which I had corrected, though too many were allowed to remain by the carelessness of the printer, but which are corrected in the subsequent edition. He goes on to state, " that the want of accuracy is inexcusable where the labour of the writer is only that of translation, and mere transcription." It is evident to me that the reviewer has never seen the original work, or that he is grossly ignorant of the French language. Had he known, that the name of each medicine is given in popular French, and that hundreds of them were to be found only in few dictionaries of terms, either ancient or modern, he would have been aware, that there was somewhat more labour to perform than mere translation or transcription. In fact the labour and trouble were so great, that I alluded to them in my preface in these words.

" The translation of this work from French into Latin and English was a troublesome undertaking. The names of many thousand medicines, seldom employed in this country, were given in popular French, and were to be rendered strictly scientific. To effect this, much research was necessary, and a vast deal of reference inevitable, which might have been avoided, were the scientific names given in the original text. It was, however, considered right, to give a literal translation in English and Latin." So much for the mere translation and transcription of the original. It was this mere transcription that led the reviewer to betray his ignorance as I shall shew hereafter. Another Arcadian of his class, denied the accuracy of my statement that thousands of medicines seldom used in this country were mentioned, (*British and Foreign Medical Review, July*, 1836), and he proved his case by referring to the index; but had he looked to the text he would have found my statement literally correct, as numerous medicines named after physicians or certain places are given, which I, of course, excluded from the index.

But your critic boldly states the whole work is inaccurate, " where ounces are put for drachms, and *vice versa*, or where weights are altogether omitted; or where the directions of one article apply to another; or where the substances blended together are incompatible to be mixed. To make these blunders the more ludicrous, a list of twenty errata is given to shew errata contained errata." In proof of this wholesale and mendacious charge the reviewer quotes two of my own prescriptions, instead of any of the original ones belonging to the text.—[*See Medico-Chirurgical Review, July* 1836.]

This honest critic selects the only misprint in a whole page and last prescription, although the word guaiacum had been printed correctly four times in the preceding formulæ, and so just is he, that he would feign to saddle the blunder on the translator. Then comes his comment against different ingredients with a view of obtaining the effects of one and all of them, as if this were not an established axiom in every work on phamacology. I would advise him very strongly to study some one of them, even my translation for example, before he again betrays his superficial acquaintance with the subject.

In the second prescription the writer not only betrays ignorance, but even malice, for he wilfully misrepresents it. The original stands thus:—

℞. Panacis v. Fol. pulv., misrepresented
Panacis *v*. Fol. pulv.
Again, Tere intime, is misrepresented
Fere intime, &c.

It is manifest that the critic did not know the medicine in the first line, and as he could not find it in any of our English works on materia medica, shews the extent of his own resources, by misspelling it. He further makes me write magnesiæ calcia, for M. calcinatæ!!—Can such contemptible conduct be called criticism?

In the preceding extracts, may I also beg to inquire, where are ounces for drachms, *vice versa*, where the weights are altogether omitted, or where the directions for the use of one article apply to another, asserted by your reviewer? That there were too many typographical errors, nearly all insignificant, I have admitted already; but that your critic should wilfully add to them to bear out his unjust strictures, is what every honest man must reprobate.

As to his wholesale condemnation of the French, German, Italian, and American formulæ in the original work, it is perfectly unnecessary to reply, for I am in no way responsible either for their efficacy or accuracy.

In comparing my translation of the Formulary of Hospitals, to Dr. Thomson's Conspectus, and M. Ratier's Formulaire of the Paris Hospitals, he again betrays his ignorance, as these works are different from each other, though excellent manuals. Thus Dr. Thomson's Conspectus of the British Pharmacopœiæ contains no hospital prescriptions, M. Ratier's is confined to the French hospital formulæ and codex, while the condemned work is a universal formulary, containing that of M. Magendie, &c. But the new Formulary of European and American Hospitals cannot be injured by such a stult as your correspondent, it has already passed through two editions in France, and the same number in this country in nine months; it is much more comprehensive and cheaper than any other English work of the kind, and has nothing to fear from incompetent critics. I offer this defence of it, having introduced it to the notice of the profession in this kingdom, and as I have determined that it shall not be unjustly criticized with impunity.

I am, Gentlemen, your obedient servant,

4, Great Queen Street, M. RYAN, M.D

St. James's Park, Westminster, Sept. 1836.

---

In the last number of this Journal we inserted a critique from a correspondent, on Dr. Ryan's work, and mentioned that we had struck out several passages and expressions which we considered to be unnecessarily acrimonious. We greatly regret that we overlooked a kind of inuendo (the real meaning of which we are still ignorant of), respecting the PUBLISHER of the work. Had we noticed this, we certainly should have immediately run our pen through it—because we are conscious that the publisher of a book, and especially of a medical book, is not at all answerable for errors or faults of the author. The critic, in this instance, has travelled out of his proper path, and we have no hesitation in expressing our regret that the passage alluded to has appeared in our pages.

---

### CHARTER OF THE UNIVERSITY OF LONDON.

The rough draught of this long expected Charter has just appeared, and we lay before our readers extracts from that part of it which relates to our own profession. We have not had time to consider it maturely, but we have little hesitation in averring that, if not materially altered, it will realize the hopes of the most sanguine medical Reformers.

"Now, know ye, that for the purpose of ascertaining, by means of examinations, the persons who have acquired proficiency in literature, science, and art, by the pursuit of such course of education, and of rewarding them by academical degrees, as evidence of their respective attainments, and marks of honour proportioned thereunto, we do, by virtue of our prerogative royal, and of our especial grace, certain knowledge, and mere motion, by these presents for us, our heirs and successors, will, grant, declare, and constitute during our royal will and pleasure, and all the persons who we may hereafter appoint to be Chancellor, Vice-Chancellor, or Fellows, as hereinafter mentioned, *one body politic and corporate*, by the name of the University of London, by which name such body politic shall have perpetual succession, and shall have a common seal, and shall by the same name sue and be sued, implead and be impleaded, and answer and be answered unto in every court of us, our heirs and successors.

And we do hereby further will and ordain, that the said body politic and corporate shall consist of one Chancellor, one Vice-Chancellor, and such number of fellows or members of the senate as we shall from time to time appoint under our sign manual; and that our trusty and well beloved the aforesaid be the first chancellor the first vice-chancellor, and the said be the first fellows and members of the senate thereof.

That whenever a vacancy shall occur in the office of chancellor of the said university, either by death, resignation, or otherwise, we will, under our sign manual, nominate a fit and proper person to be the chancellor instead of the chancellor occasioning such vacancy.

That the office of Vice-Chancellor of the said University shall be *an annual office*, and the Vice-Chancellor hereinbefore named shall, at the expiration of one year from go out of office, and the said Fellows or Members of the senate shall, at a meeting to be holden by them for that purpose, on some day within a month before the expiration of the said office, of which due notice shall be given, elect one other fit and proper person to be the Vice-chancellor of the said University, and so from time to time annually; or, in case of the death, resignation, or other avoidance of any such Vice-Chancellor before the expiration of his year of office, shall, at a meeting to be holden by them for that purpose as soon as conveniently may be, of which due notice shall be given, elect some other fit and proper person to be Vice-Chancellor for the remainder of the year, in which such death, resignation, or other avoidance shall happen; such person to be chosen from among themselves by the major part of the Fellows present at such meeting, and to be approved of by the Chancellor of the said University for the time being.

That we reserve to ourselves to be the visitor of the said University of London, with authority to do all those things which pertain to visitors as often as to us shall seem meet.

That the Chancellor, Vice-Chancellor, and Fellows for the time being, shall have the entire management of and superintendence over the affairs, concerns, and property of the said University; and in all cases unprovided for by this our charter, it shall be lawful for the Chancellor, Vice-Chancellor, and Fellows, to act in such manner as shall appear to them best calculated to promote the purposes intended by the said University, and the said Chancellor, Vice-Chancellor, and Fellows shall, have full power from time to time to make and also to alter any by-laws and regulations (so as the same be not repugnant to the laws of our realm, or to the general objects and provisions of this our charter) touching the examinations for degrees, and the granting of the same, and touching the mode and time of convening the meetings of the Chancellor, Vice-Chancellor, and Fellows, and in general touching all other matters whatsoever regarding the said University; and all such by-laws and regulations when reduced into writing, and after the common seal of the said University has been affixed thereto, shall be binding upon all persons members thereof, and all candidates for degrees to be conferred by the same, all such by-laws and regulations having been first submitted to one of our principal Secretaries of State, and approved of and countersigned by him.

That all questions which shall come before the Chancellor, Vice-Chancellor, and Fellows, shall be decided by the majority of the members present, and the chairman at any such meeting shall have a vote, and in case of an equality of votes, a second or casting vote.

That no question shall be decided at any meeting, unless the Chancellor, or Vice-Chancellor, and five Fellows, or, in the absence of the Chancellor and Vice-Chancellor, unless six Fellows at the least shall be present at the time of such decision.

That at every meeting of the said Chancellor, Vice-Chancellor, and Fellows, the Chancellor, or in his absence the Vice-Chancellor, shall preside as chairman, or, in the absence of both, a chairman shall be chosen by the members present, or the major part.

That the said Chancellor, Vice-Chancellor, and Fellows for the time being, shall have full power from time to time to appoint, and, as they shall see occasion, to remove all necessary examiners, officers, and servants of the said University.

That once at least in every year the said Chancellor, Vice-Chancellor, and Fellows, shall cause to be held an examination of candidates for degrees, and on every such examination the candidates shall be examined either by examiners appointed for the purpose from among the fellows by the said Chancellor, Vice-Chancellor, and Fellows, or by other examiners so to be appointed; and that on every such examination the candidates shall be examined in as many branches of general knowledge as the said Chancellor, Vice-Chancellor, and Fellows, shall consider the most fitting subjects of such examination.

And whereas it is expedient to extend the benefits of colleges and *establishments* already instituted, or which may be hereafter instituted, for the promotion of literature and science, whether incorporated or *not incorporated*, by connecting them for such purposes with the university created by this our royal charter. We do hereby further will and ordain that all persons shall be admitted as candidates for the respective degrees of Bachelor of Arts, Master of Arts, Bachelor of Laws, or Doctor of Laws, to be conferred by the said University of London, on presenting to the said Chancellor, Vice-Chancellor, and Fellows, a certificate from any of the institutions hereinafter mentioned, to the effect that such candidate has completed the course of instruction which the said Chancellor, Vice-Chancellor, and Fellows, by regulation in that behalf shall determine.

That such certificates as aforesaid may be granted from our college called London University College, or from our college called King's College, or from such other institution, corporate or unincorporated, as now is or hereafter shall be established for the purposes of education, whether in the metropolis or elsewhere within our United Kingdom, and as we, under our sign manual, shall hereafter authorize to issue such certificates.

And for the purpose of granting the degrees of Bachelor of Medicine, and Doctor of Medicine, and for the improvement of medical education *in all its branches, as well in medicine as in surgery, midwifery, and pharmacy*, we do further hereby will and ordain that the said Chancellor, Vice-Chancellor, and Fellows, shall from time to time report to one of our principal Secretaries of State what appear to them to be the medical institutions and schools, *whether corporate or incorporated*, in this our metropolis, or in other parts of our United Kingdom, from which, in the judgment of the said Chancellor, Vice-Chancellor, and Fellows, it may be fit and expedient to admit candidates for medical degrees, and on approval of such report by our said Secretary of State, shall admit all persons as candidates for the respective degrees of *Bachelor of Medicine and Doctor of Medicine* to be conferred by the said University, on presenting to the said Chancellor, Vice-Chancellor, and Fellows, a certificate from any such institution or school, to the effect that such candidate has completed the course of instruction which the said Chancellor, and Fellows, by regulation in that behalf shall determine; and it shall be lawful for the said Chancellor, Vice-Chancellor, and Fellows, from time to time, with the approval of one of our principal Secretaries of State, to vary, alter, and amend any such reports, by striking out any of the said institutions or schools included therein, or by adding others thereunto.

That the said Chancellor, Vice-Chancellor, and Fellows, shall have power to confer the several degrees of Bachelor of Arts, Master of Arts, Bachelor of Laws, Doctor of Laws, Bachelor of Medicine, Doctor of Medicine, and that such reasonable fees shall be charged for the degrees so conferred as the said Chancellor, Vice-Chancellor, and Fellows, with the approbation of the commissioners of our Treasury shall from time to time direct; and such fees shall be carried to one general fee-fund for the payment of the expences of the said University, under the directions and regulations of the commissioners of our Treasury, to whom the accounts of income and expenditure of the said University shall once in every year be submitted, which accounts shall be subject to such examination and audit as the said commissioners may direct.

That at the conclusion of every examination of the candidates the examiners shall declare the name of every candidate whom they shall have deemed to be entitled to any of the said degrees, and the departments of knowledge in which his proficiency shall have been evinced, and also his proficiency in relation to that of other candidates, and he shall receive from the said Chancellor a certificate, under the seal of the said University of London, and signed by the said Chancellor, in which the particulars so declared shall be stated.

Provided always, that all by-laws and regulations made from time to time touching the examinations of candidates and granting of degrees, shall be submitted for the consideration of one of our principal Secretaries of State, to be approved of by him.

And lastly, we do hereby for us, our heirs, and successors, grant and declare that these our letters patent, or the enrolment or exemplification thereof, shall be in and by all things valid and effectual in law, according to the true intent and meaning of the same, and shall be construed and adjudged in the most favourable and beneficial sense for the best advantage of the said University, as well as in all our courts as elsewhere, notwithstanding any non-recital, mis-recital, uncertainty, or imperfection, in these our letters patent. In witness, &c."

Thus then, it is clear that all proper and recognized schools of medicine will be entitled to send up candidates for degrees, after passing through the regular curricula, to be examined at the metropolitan board. We appeal to every unprejudiced reader whether this Charter if confirmed by Parliament, does or does not involve much of the ridiculed "ONE FACULTY," which we have so long advocated? Candidates are to comply with a certain curriculum fixed by the London Council—they are to be examined in *physic*, *surgery*, *midwifery*, and *pharmacy*—and they are to have only one *kind* of degree—either Bachelor or Doctor of Medicine! This is very like "ONE FACULTY." The candidate may obtain his knowledge in town or country—provided always (which we have invariably advocated) that the school be properly calculated to afford adequate medical instruction. What then becomes of the College of Physicians—of Surgeons—or of Apothecaries? They must answer this question themselves. If the candidate be examined in all branches of medical science and obtain a degree, for what purpose will he repair to Pall Mall East, Lincoln's-inn-fields, or Blackfriars? Perhaps for the pleasure (for there be small honour) of paying forty or fifty pounds—the medical candidates being, as is well known, exuberant in funds!—"*Our* college, called 'LONDON COLLEGE'—and *our* college, called 'KING'S COLLEGE,'" will have no more privileges than Guy's or St. Thomas's—Bartholomew's or St. George's—or, in fact, any other recognized school of medicine! The same applies to Oxford and Cambridge—if indeed these seminaries be recognized as medical schools. In fine, this Charter goes as far as our most sanguine hopes could have anticipated, provided it be confirmed by Parliament, so as to entitle the diplomatists to practise the branches on which they have been examined.

### UNFERMENTED BREAD.

Dr. Whiting, of Kennington, has discovered a mode of baking bread, from salt, water, and flour, without fermentation, without yeast or barm. The salt is formed during the manufacture of the bread by the combination of bicarbonate of soda and dilute muriatic acid, so as to throw the dough into a cellular structure, by means of the carbonic acid gas evolved during the process.

In Dr. Whiting's experience this unfermented bread has proved very serviceable in dyspepsy—in disposition to form urinary concretions, &c. We have now been using it ourselves for ten days or a fortnight, and find it to agree remarkably well with the stomach. We anticipate important advantages from its general use. It is quite as pleasant as any other bread that we have eaten. We procure our supply from Mr. Tate, 58, High Holborn.

### DR. BRIGHT ON DISEASED KIDNEYS.

6, *Union Street, Southwark, July 1st*, 1836.

SIR,—In the number of the Medico-Chirurgical Review of this day, I observe a critique upon a paper by Dr. Bright, on diseased kidneys, published in the Guy's Hospital Reports, in which you have spoken of remarks upon urine by Dr. Barlow, *of Bath*, and also have stated that he is about to publish some further observations upon the same subject in a future number of these Reports.

Now, Dr. Barlow, *of Bath*, is indeed mentioned by Dr. Bright in the paper in question, but he is never there spoken of except with the words "of Bath" after his name, which were inserted by way of distinction from myself, who am the person alluded to by Dr. Bright, as Dr. Barlow, or Dr. G. H. Barlow, and who am, moreover, one of the editors of the reports.

Now, Sir, the short article which I have already inserted in the reports (and which you have ascribed to my namesake), contains opinions which have been controverted by some, and the remarks which I intend shortly to publish, may be open to further objections; it is, therefore, hardly fair upon Dr. Barlow, of Bath, to make him answerable for my errors, any more than it is just towards the public to offer to them under the sanction of his well-earned reputation the possibly erroneous opinions of myself.

You will, I am sure, pardon me for troubling you with this communication, as I consider that it is due to yourself to afford you an opportunity of correcting an error into which you have inadvertently fallen.

I am, Sir, your obedient servant,
G. H. BARLOW, M.A. and L.M.
Inceptor Candidate of the College of Physicians.

---

### GOLD POINTED LANCET.

*Jersey, 9th July*, 1836.

Dear Sir,—I have invented a lancet with a gold point, for inoculation, and its advantages are,—First, it can at all times be introduced under the cuticle, without extracting blood, a circumstance always calculated to defeat the success of the operation.—2ndly. It cannot rust, and is to be cleaned with facility.—3rdly. It can be kept charged with lymph for a much longer period.—4thly. The idea of operating with a gold instrument, will be always much more pleasing to mother and child.

Believe me to remain, with much esteem, dear Sir, your's &c.
PRIMROSE LYON, Surgeon, R.N.

---

### NEW AND NUTRITIOUS ALIMENT.

[*From Dr. Ryan's Journal.*]

"I have been favoured with a small box of a new and very delicious aliment, by Dr. Waterson, of St. Kitt's, who has also furnished me with the following account of it. This substance is the pith of the roots of the *Canna Coccinea* when eight months old. The preparation of it is more tedious and troublesome than that of arrow-root. The canna coccinea flowers every month, and this has led the French to term it, ' tous les mois ;' and a corrupt English translation, in popular use, is ' toulemong.'

'You will find,' continues Dr. Waterson, ' by using it in your own family, and by analysing it, that it differs most materially from arrow-root; and I consider it, as a diet for the sick, very far superior to sago, tapioca, arrow-root, and gruel. My own experience makes me satisfied, that it is a most nutritious diet, easily digestible, and is, consequently, invaluable in diets for infants and children, and is eminently adapted for persons of dysenteric, diarrhœal, and consumptive habits, because there is, in my belief, less acid in it than any other farinaceous food with which I am acquainted. I have never known it turn sour upon the stomach. It is in great demand for fever diet, thickening soups, and making puddings, as a little goes a great way. It is the ordinary food of the dyspeptic, and in enemas it is truly serviceable for allaying the effects of acrid bile on the coats of the rectum, and for sheathing them in cases of abrasion or inflammation.'

Agreeable to the request of my correspondent, I have tried the tous les mois in my own family. It makes a more consistent and delicious jelly than arrow-root, and is, in my opinion, far superior to it or any other farinaceous powder used in this country as an aliment for infants, inva ids, or convalescents. It is prepared like arrow-root, and about half the quantity makes a thicker jelly. I have given it to my own children, used it at my own table, and presented quantities of it to my friends. It deserves the high praise bestowed upon it by Dr. Waterson, and cannot fail to be patronized by the medical profession and the public.

It is cultivated by Mr. Olpherts, a magistrate, at St. Kitt's, and a scientific agriculturist.

It may be procured in London, from Mr. Pearce, Pedler's Acre, Lambeth.

M. RYAN, M.D.
Great Queen Street, St. James's Park, Westminster, Aug. 9, 1836.

☞ Dr. J. Johnson has been using the nutriment above-mentioned, for more than a month past, every day; and can confirm the statements of Drs. Waterson and Ryan in respect to the lightness, nutritious quality, and indisposition to acidity of the article in question.

***Extracts from a Petition to Parliament of the Medical Professors of the Andersonian University, for an Extension of University Reform.***

SHEWETH,

That the Monopoly of Teaching is enforced with different degrees of rigour and under various modifications in the different Scotch Universities, and your Petitioners conceive that they will best convey an adequate idea of the oppressive nature of this monopoly, by confining their statements to the monopoly asssumed by the University of Glasgow.

According to the charter all graduates are constituted teachers, and in the diplomas which your Petitioners, or most of their number, hold of the University, the privilege of teaching is conferred in the most explicit terms; but on their attempting to exercise that privilege, they are informed, by the rejection of their certificates, that the Principal and professors do not consider as obligatory *a verbal promise solemnly given, and confirmed by a written document ! ! !*

That the monopoly in teaching assumed by the Principal and Professors of the University of Glasgow, consists in this, that in conferring degrees in Medicine, for which a certain curriculum of study on the part of the candidate is required, they receive no certificates of instruction but from Professors in Universities, or from teachers in London and Dublin, who, being at a distance from themselves, cannot enter into any immediate competition with them, while they reject the certificates of all other teachers, however well qualified, and even although holding their own diplomas conferring the privilege to teach.

That in consequence of this monopoly, all students who intend to take degrees in Medicine are compelled to attend the Classes of the University, even in the case which may possibly occur, when that attendance can only be regarded as a mis-spending of time. The Professors are thus secluded from all competition, and deprived of most powerful motives, which might stimulate them to exertion. The hurtful consequences of such a system to the rising generation of Medical men, to Medical Science, to the city of Glasgow, to the public at large, and even to the University, by which the system is enforced, are too obvious to require commentary.

That the admission of the certificates of teachers in London and Dublin, while the certificates of your Petitioners are rejected, is at once invidious and unjust. A privilege is thus given by the Principal and Professors to total strangers, which is denied to teachers in this city, most of whom are their own *alumni*, and whose qualifications to teach they can easily ascertain. Nor is it from any distrust of the qualifications of your Petitioners that this privilege is withheld, seeing that any of them would at once receive it on shifting his abode to London or Dublin.

That the concession made to the Teachers in London and Dublin seems to proceed from *the desire of increasing the number of those who take degrees from the University.* To the same motive must be referred the extraordinary course which the Principal and Professors have adopted, inasmuch as while all other Medical Boards throughout the kingdom have been raising the standard of qualification in the Medical Student, *the members of the University of Glasgow have lowered the standard which they require.*

That the University of Glasgow has for some years past assumed a power which no other University in this country ever before pretended to, that of granting licences to practise Surgery.

Last of all, and chiefly, your Petitioners would beg to call the attention of your Right Honourable House to the circumstance, that a monopoly in Teaching does not differ from a monopoly in any other branch of trade. With respect to the latter, it is well known that the profits of the monopolist are paid out of the pockets of those engaged in competing with him in that branch of trade to which his monopoly relates. On the very same principles, it is demonstrable that the whole profits which the Professors of the University derive from their monopoly are paid solely by your Petitioners, and by the other private teachers in this city.

Robert Hunter, M.D. Professor of Anatomy and Physiology.
Alexander J. Hanney, M.D. Professor of the Theory and Practice of Physic.
Andrew Buchanan, M.D. Professor of Materia Medica.
Thomas Graham, Professor of Chemistry.
James Brown, M.D. Professor of Midwifery.
George Watt, Professor of Medical Jurisprudence.

